# A review on pathobiology of circulating tumour plasma cells: The sine qua non of poor prognosis in plasma cell neoplasms

**DOI:** 10.32604/or.2024.055154

**Published:** 2025-04-18

**Authors:** PRATIBHA SUKU, AISHWARYA DASH, ARAVIND RADHAKRISHNAN, PANKAJ MALHOTRA, MAN UPDESH SINGH SACHDEVA

**Affiliations:** 1 Department of Hematology, Postgraduate Institute of Medical Education and Research (PGIMER), Chandigarh, P.O. Box 160012, India; 2 Department of Clinical Hematology and Medical Oncology, Postgraduate Institute of Medical Education and Research (PGIMER), Chandigarh, P.O. Box 160012, India

**Keywords:** Circulating Plasma Cells (CPCs), Multiple Myeloma (MM), Flow cytometry, Circulating Tumor Plasma Cells (CTPCs), Microenvironment, Monoclonal gammopathy

## Abstract

Circulating plasma cells (CPCs) in patients of plasma cell neoplasm have been an area of intense research in recent decades. Circulating tumor plasma cells (CTPCs) might represent a sub-clone of tumor cells that have exited into peripheral blood as a result of the dynamic interactions between the bone marrow (BM) microenvironment and neoplastic plasma cells. Chemokine receptors like chemokine receptor 4 (CXCR4) and integrins are known to play a role in homing and migration of plasma cells (PCs). The hypoxic microenvironment in the BM niche also contributes to their circulation through various mechanisms. In addition, the CCL3–CCR1 axis probably competes with the retention signals from the CXCR4–α4β1 (VLA-4) interaction and actively promotes the exit of PCs from the BM. CTPCs, even in extremely low numbers, can be detected and quantified by high-sensitivity techniques like multi-color flow cytometry and next-generation sequencing. High load of CTPCs noted in patients of plasma cell neoplasm; monoclonal gammopathy of undetermined significance (MGUS), smoldering multiple myeloma (SMM), multiple myeloma (MM) is a strong predictor of shorter progression free survival (PFS) as well as overall survival (OS). In newly diagnosed patients of MM, a load of CTPCs correlates with the outcomes, i.e., OS and PFS. With more studies collaborating on the results of previous reports, assessment of the burden of CTPCs may become a complimentary approach for non-invasive risk stratification of MM patients and evaluating the response to therapy. Future research on larger cohorts and longer follow-ups may help to improve the existing staging system by incorporating the load of CTPCs as one of the prognostic indicators. Further studies based on isolation and genetic characterization of CTPCs may help in understanding the pathophysiology of the progression of the disease and may open avenues for newer treatment modalities. This review discusses the pathobiological aspects leading to circulation of neoplastic/tumor plasma cells in peripheral blood and provides a summary of research work done in last two decades on its prognostic importance in various plasma cells neoplasms.

## Introduction

Multiple myeloma (MM) is a malignant plasma cell (PC) neoplasm and represents the second most common cancer of B-cell origin in the elderly population. The age standardized rate (ASR) of MM incidence was 1.78 (95% UI 1.69–1.87) per 100,000 people globally and mortality was 1.14 (95%UI 1.07–1.21) per 100,000 people globally in 2020 [1,2]. The median age at the time of diagnosis is commonly found to be between 72–74 years; however various studies indicate that a small percentage of cases occur in individuals younger than 40 years [3–5]. The diagnosis of MM involves multiple disciplines and includes an assessment of clinical features, biochemical parameters, and radiological and histo-pathological findings for a complete workup. The overall survival of the patients depends on numerous variables, some of which include the biological characteristics of tumor cells, patient-related features such as overall health status, and drug-dependent factors like treatment toxicities [1]. Clinical features associated with MM include hypercalcemia, renal failure, anemia, or bone lesions (CRAB criteria). There may be other signs and symptoms due to bone marrow suppression caused by infiltration of tumor cells and as a consequence of over-production of monoclonal proteins.

MM is a biologically heterogeneous neoplasm with a spectrum of phenotypic variations, such as the clinical features at the time of presentation, the course of the disease, and overall survival (OS) amongst its patients. The survival outcomes and response to therapy depend on many prognostic factors incorporated into clinical practices. Scoring systems were developed to address MM clinical heterogeneity for assessing individual prognosis [6,7]. These systems have evolved over decades and some of the well-known scoring systems along with their main parameters are listed below:

- Salmon & Durie (SD) staging system–anemia, osteolysis, and renal failure
- International Staging System (ISS)–serum levels of β2-microglobulin and albumin
- Revised International Staging System (R-ISS)–ISS, LDH, and high-risk cytogenetic abnormalities (CA)
- Revised International Staging System (R-ISS2)–RISS and 1q21 gain [8–11]

These scoring systems are mostly used at the time of diagnostic evaluation of patients to assist in management decisions [4]. In addition to the above, the role of minimal residual disease analysis, especially at the end of induction, is an important indicator of the depth of treatment response [12].

Bone marrow aspiration and trephine biopsy remain indispensable for workup and further management of MM patients. It provides an estimate of tumor burden and provides material for axillary techniques beyond light microscopy, like immunohistochemistry, fluorescent *in-situ* hybridization (FISH), flow cytometry, and other molecular techniques including state of art of next generation sequencing (NGS) [13,14]. These are crucial for diagnosis as well as prognostication of MM. The International Myeloma Working Group (IMWG) consensus requires either BM aspirate and/or trephine biopsy for PC estimation. However, it is important to note that the PC counts may not be accurate due to their uneven distribution in BM, or due to dilution by peripheral blood [15]. Estimates of tumor burden are important prognostic indicators and hence new approaches for accurate determination may result in better outcomes.

Precursors of MM like SMM are not treated but kept under observation. MM is conventionally treated with multiple drug therapy, mostly a combination of bortezomib, lenalidomide/cyclophosphamide, dexamethasone (VRd/VCd) and some centers may add daratumumab (anti-CD38) upfront [12]. The response to therapy is mostly associated with overall survival (OS) as per RISS categorization.

The primarily affected tissue in MM is the BM, even though studies consistently demonstrate that a significant percentage of patients also have peripheral blood (PB) involvement [16].

The pathobiological function of myeloma cells in peripheral blood circulation has been intensively studied in the past but remains a matter of debate. Studies have categorically shown that the presence of CPCs in newly diagnosed patients of MM is a strong predictor of poor prognosis [17–20]. The presence of CPCs also indicates a worse outcome in patients undergoing autologous stem cell transplantation (ASCT) [21–23]. A higher load of CPCs in individuals of monoclonal gammopathy of unknown significance (MGUS) and smoldering multiple myeloma (SMM) indicates a higher risk of progression to symptomatic MM [24]. Overall, the burden of CPCs in individuals with a plasma cell neoplasm represents a prognostic factor related to the biological characteristics of the disease [25–27]. Studies in the past decade have tried to give a cutoff for a load of CTPCs that can help stage patients.

The authors aim to review the literature and discuss important aspects of extensive research work carried out in last two decades, including the mechanisms underlying circulation of tumor plasma cells in-and-out of bone marrow and the patho-biology of CTPCs; the basic principles, advantages and limitations of frequently used techniques for detection and quantification of CTPCs; and the role of CTPCs in prognostication of pre-malignment plasma cell neoplasms as well a symptomatic multiple myeloma.

## Pathophysiology of Circulating Tumor Plasma Cells (CTPCs)

CTPCs have been generally accepted to reflect the biology of neoplasm representing as a marker, related to, but not necessarily dependent on the overall tumor load [22,27]. The patho-biological role of CTPCs in plasma cell neoplasms has been evaluated in many studies but not completely understood [28,29]. Studies indicate that CTPCs represent a distinct subclone of BM PCs with a quiescent profile and a more immature phenotype [22,30–32]. Several studies have attempted to show a correlation between the burden of myeloma cells in BM and a load of CTPCs, however, the findings have not been confirmatory [33,34]. There have been attempts by multiple groups to evaluate the bi-directional interactions between tumor cells and the micro-environment which influence the homing, retention and migration of myeloma cells. Certain events like the hypoxic micro-environment promote angiogenesis by promoting the secretion of vascular endothelial growth factor (VEGF) which also enhances the growth of tumor. In addition, there is down-regulation of the expression of many adhesion molecules on myeloma cells, such as CD56, CD117, CD81 and CD138 [28,35,36]. Further, there is a loss of expression of certain integrins like CD49d, CD49e, CD11a, CD11c & CD29 and of some of the activation-related antigens like CD38 and CD27. However, the pathobiology of the egress of myeloma cells from bone marrow in the circulation is intricately associated with bone marrow microenvironment and requires an understanding of the complex interactions.

### Bone marrow microenvironment and myeloma cells

The “myeloma cell niche” or its microenvironment in bone marrow comprises both the non-cancer cells and their stroma. Both these components influence growth, metastatic potential and response to therapy of myeloma cells. The stroma or the extracellular matrix is composed predominantly of fibronectin, laminin, collagen type I & IV, and glycosaminoglycans like hyaluronan, heparan sulphate and chondroitin sulfate [37]. The cellular components known to play an important role in myeloma-pathobiology include bone marrow mesenchymal stromal cells (BMMSCs), osteoblasts, osteoclasts, endothelial cells, pericytes, adipocytes, and immune cells including macrophages, dendritic cells, lymphocytes, and myeloid-derived suppressor cells (MDSCs) [38]. The myeloma cells express adhesion molecules on their cell membrane which can bind with the components of the extracellular matrix, like fibrinogen & laminin bind to β1-integrins, hyaluron with CD44 and collagen-I binds to syndecan (CD138) [38].

The interaction of VLA-4 (β1-integrin) with fibronectin is one of the early steps in homing of the plasma cells in bone marrow. The secretion of SDF-1 (CXCL12) from the bone marrow microenvironment contributes to the upregulation of VLA-4 expression on myeloma cells which increases their ability to bind to the extracellular matrix [39]. In addition, adherence to fibronectin also seems to increase chemoresistance amongst the tumor cells, likely via NF-κB pathway. Out of all the cellular components of the “myeloma cell niche”, BMMSCs stand-out for their central role in plasma cell survival and growth. Several sub-types of BMMSCs have been identified, i.e., CXCL12-abundant reticulin (CAR) cells, leptin-receptor (LPR^+^) expressing stromal cells, neural markers NG2 and Nestin expressing stromal cells. These BMMSCs are known to play an important role in regulation of hematopoietic stem cell homeostasis.

### Chemokine and other cells involved in MM cell trafficking

Studies have described various MM cell trafficking events which enable the tumor cells to enter & inhabit specific BM niches, and in the course of the disease, egress BM to re-circulate, leading to extramedullary disease. Notably, CXCR4, a chemokine receptor expressed on the majority of plasma cells, interacts with its ligand CXCL12, a chemokine secreted by cells constituting the tumor microenvironment, especially the BMMSCs. CXCL12 has three isoforms: α, β, and γ, all of which can interact with heparin sulfate and extracellular matrix. Its interaction with the extracellular matrix suggests that rather than freely floating, CXCL12 is immobilized in BM. The γ isoform is the most abundant in the BM milieu. It has an extended C-terminus that binds with a higher affinity to heparin sulfate as compared to other isoforms, promoting myeloma cell adhesion [40]. The CXCR4-CXCL12 interaction mediates homing and lodging, as well as retention of both normal and malignant plasma cells into the BM [41–43]. The very initial step of the attachment of myeloma cells to BM microvasculature is contributed by P-and E-selectins and their ligands. P-selectin glycoprotein ligand-1 (PSGL-1), which is expressed on the surface of myeloma cells interacts with the endothelial cells of the BM micro-vessels [44]. This mediates the rolling of plasma cells on the P-selectins expressed on the endothelial cells. Further, the CXCL12-CXCR4 interaction on the surface of MM cells upregulates the activity of the α4β1 integrin, enhancing binding to its ligand vascular cell adhesion molecule 1 (VCAM-1), which is also expressed on the micro-vessels of the BM [45]. This adhesive event is crucial in myeloma cell trafficking into the milieu of bone marrow. In addition to the α4β1-VCAM-1 interaction, the other significant players in myeloma cell trafficking are α4β7 integrin which interacts with MAdCAM-1 and CD44 which interacts with fibronectin [46,47]. The interaction of α4β1 integrin with VCAM-1 and fibronectin triggers MM cell signaling for the production of interleukin-6 (IL-6). The interaction of IL-6 in the BM microenvironment and IL-6 receptor present on myeloma cells stimulate its growth and development. IL-6 also upregulates CD44 expression on the surface of MM cells. Hence, this IL-6 & CD44 loop, leading to higher expression of each other, causes enormous stimulation of MM-growth signals. BMMSCs secrete many other factors which affect MM cell growth, i.e., stem cell factor (SCF), insulin-like growth factor (IGF-1), vascular endothelial growth factor (VEGF), hepatocyte growth factor (HGF) and macrophage inflammatory protein-1α (MIP-1α). However, inside the myeloma cell niche, two soluble mediators, i.e., a proliferation-induced ligand (APRIL) and B-cell activation factor (BAFF) interact with B-cell maturation antigen (BCMA) on the surface of MM cells, play an important role in survival and proliferation of tumor plasma cells. APRIL, known to be secreted by immune cells in the BM milieu surrounding plasma cells, has also been studied for its role in PC mobility inside the bone marrow. Animal studies have shown that the plasma cells proliferate and grow in APRIL-rich regions of BM. The increase in PC-numbers causes a decreased availability of APRIL, which in turn causes PCs to move to a separate neighboring BM niche with better availability of APRIL. This process seems to be an important factor for the intermittent movement of PCs inside the BM [43,48–50].

### Egression of MM cells from BM to PB

**Figure 1:**
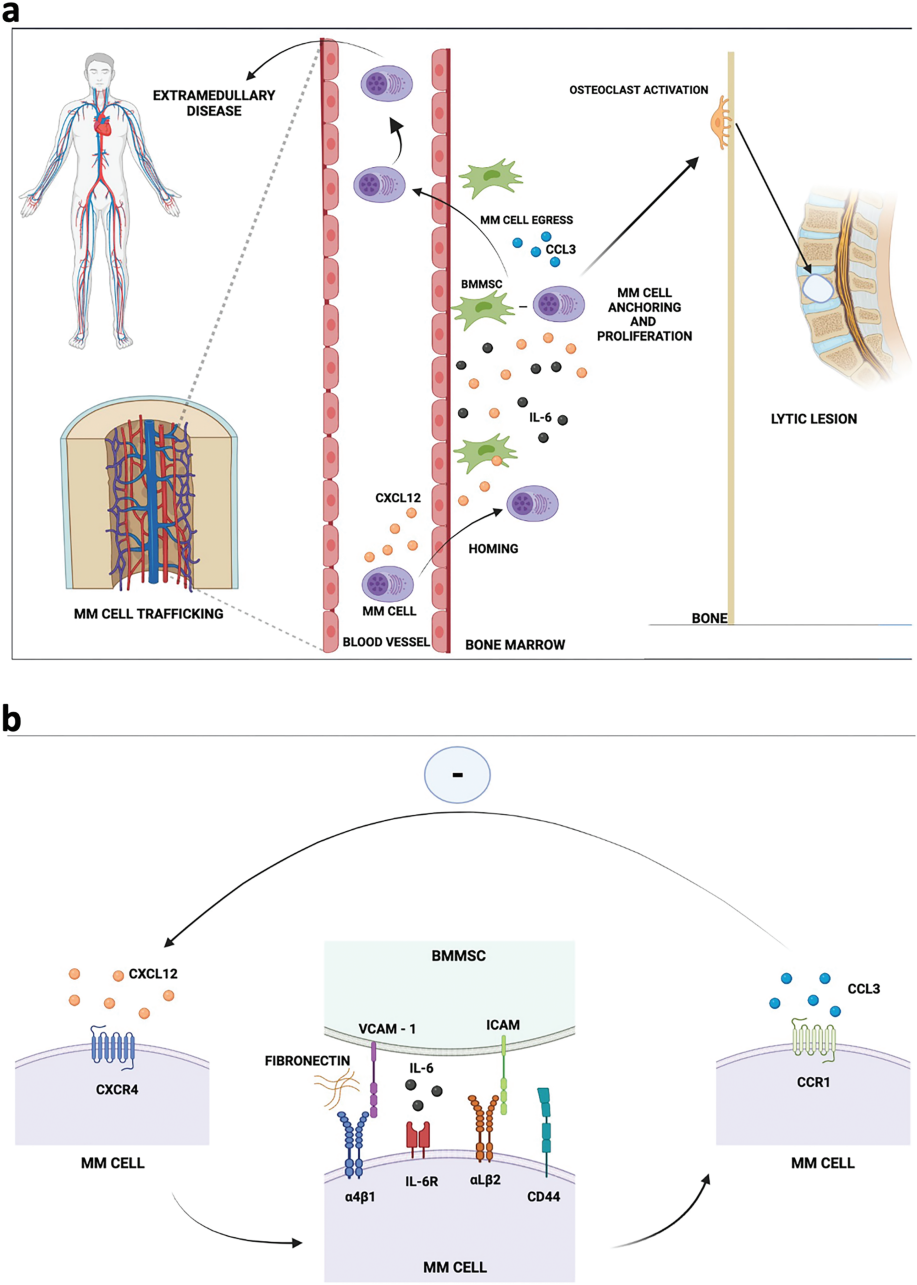
Multiple Myeloma (MM) cell trafficking events and its interaction with bone marrow mesenchymal stromal cells (BMMSC); (a) the myeloma cells in peripheral circulation home to bone marrow under the influence of CXCL12 chemokine secreted by cells comprising the bone marrow microenvironment. The interactions of myeloma cells with BM microenvironment are responsible for retention and growth of myeloma cells, which further influence activation of osteoclasts, causing lytic lesions. The myeloma cells exit into peripheral blood circulation under influence of CCL3 which may lead to extramedullary disease; (b) highlights the interaction of MM cells with BMMSCs: CXCR4 on surface of myeloma cells interacts with its ligand CXCL12 for homing into BM, this induces interaction with BMMSC and upregulation of adhesion molecules responsible for retention and growth of myeloma cells. Further, the interaction between CCR1 receptor on MM cells and its ligand CCL3 has a negative influence on CXCR4 & CXCL12 axis, causing the myeloma cells to exit BM. *The figure has been created on BioRender.com*.

During the late stages of growth of MM, the myeloma cells tend to become independent of growth signals provided by the BM milieu and there are alterations in their interactions with the BM microenvironment. The myeloma cells downregulate the surface expression of CXCR4 and instead start expressing another chemokine receptor, i.e., CCR1, which is known to enhance the egress of tumor cells from BM [51]. The increased surface expression of CCR1 leads to higher interaction with its ligand CCL3. This CCR1-CCL3 interaction inhibits myeloma cell migration towards CXCL12. This prevents interaction of CXCR4 with CXCL12 which further reduces α4β1 (VLA-4) and VCAM-1 activation, promoting myeloma cell exit from bone marrow. Animal studies have shown that blockade of CXCR4 or CXCL12 or VLA-4 contributes to the movement of plasma cells outside the BM. Processes like infection, inflammation and aging also bring about changes in the expression profile of these molecules and contribute to the egress of plasma cells. The exit of tumor plasma cells in peripheral blood circulation causes the extramedullary spread of MM [52,53]. Fig. 1a and b summarizes the main molecular events involved in homing and circulation of CTPCs.

## Techniques for Assessment of CPCs

### Conventional cytology

This was the earliest method used for the detection of CPCs by use of light microscopic examination of peripheral blood smear. It is a simple, fast, easily available and cost-effective method present in every diagnostic center. However, the sensitivity of detecting CPCs on peripheral blood film light microscopy is 10^−2^ and is much lower than presently available high-sensitivity techniques. In addition, morphological assessment of CPCs is not helpful in distinguishing normal versus tumor plasma cells [51]. Notably, the criteria for diagnosis of plasma cell leukemia, i.e., ≥5% CPCs, is based primarily on morphological assessment of peripheral blood smear.

### Multiparametric flow cytometry (MFC)

**Figure 2:**
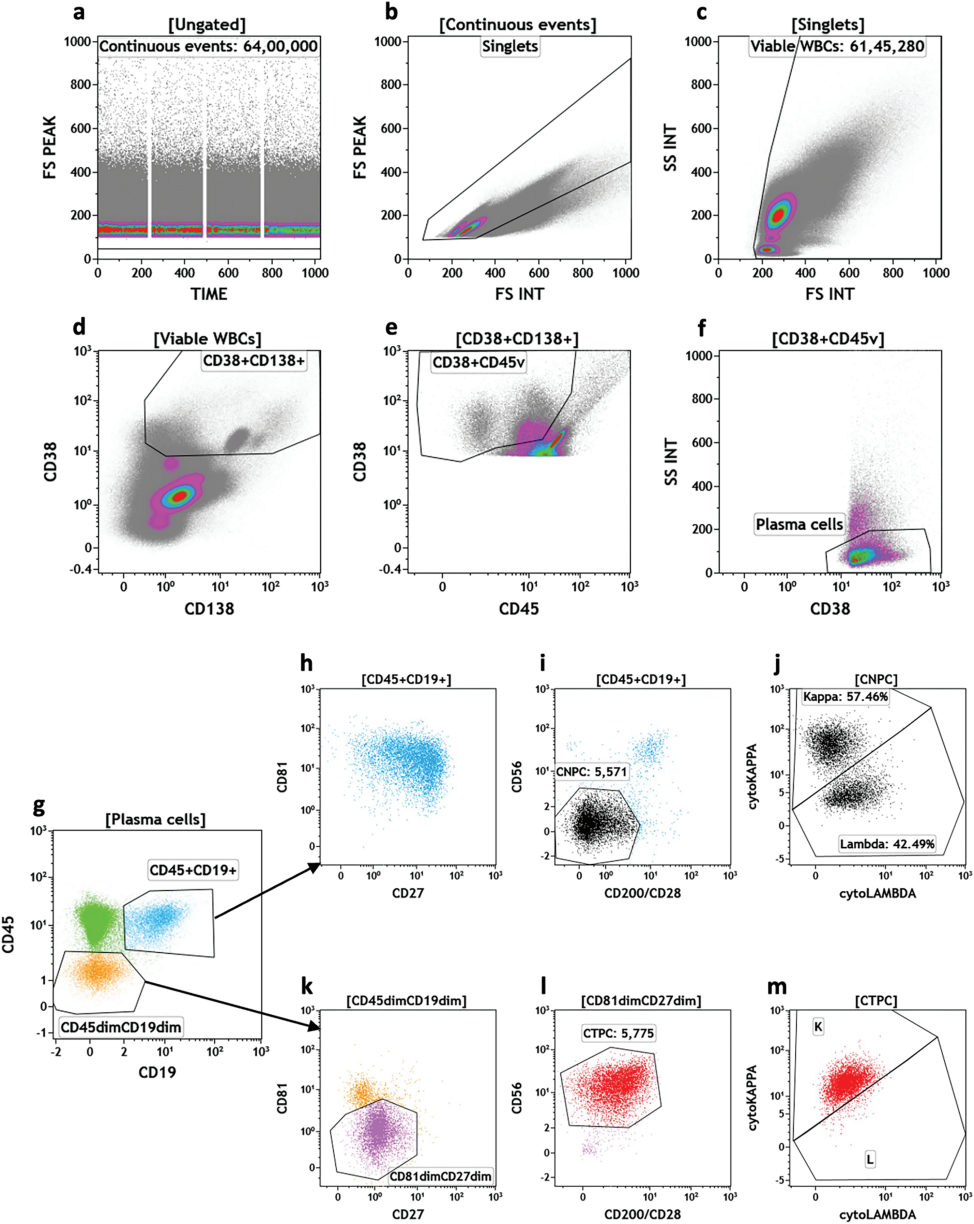
Representative bi-axial dot-plots of flow cytometric data from peripheral blood sample of a patient of MM diagnosed in our institute. a, b, c: sequential gates for inclusion of uniformly acquired continuous events, non-aggregated and non-debris viable cells, respectively; d: gating of CD38 & CD138 variably-expressed events; e: gating of CD38 positive and CD45 variable (dim to moderately-bright) events; f: gating of nicely clustered CD38 positive & SSC-low events which include the CPCs; g: plasma cells divided in two distinct populations, i.e., CD 45 & CD19 positive events (blue dots) and CD45 & CD19 dim events (orange dots), in addition, non-plasma cells (green dots) have been excluded; h: CD45+CD19+ events displayed on CD81 *vs*. CD27 plot and show bright expression of CD81 and CD27; i: Circulating Normal Plasma Cells (CNPCs) have been gated based on absence of CD56 and CD200/CD28 expression (black dots); j: CNPCs divide into cytoplasmic kappa light chain and cytoplasmic lambda light chain expressing cells; k: CD45dimCD19dim events have been displayed on CD81 *vs*. CD27 plot and a cluster of cells with absence of CD81 and CD27 have been gated (violet dots); l: CTPCs have been gated based on CD56 & CD200/CD28 positive expression levels (red dots); m: CTPCs show cytoplasmic kappa light chain restriction. *The numbers mentioned along with gated population in some of the plots represent the number of events recorded for the respective gated population.

MFC of peripheral blood is a highly sensitive, affordable and widely available technique. It can be easily used for the assessment of disease burden outside of the BM. This technique is rapid, consistent, and directly quantitative. The panel of markers generally includes antibodies against CD38, CD138, and CD45 for gating plasma cells which are then analyzed for expression of surface markers like CD19, CD56, CD200, CD117, CD81, CD10, CD20, CD28, CD27 [12,54]. A deviation from the normal expression profile of these markers is used to characterize and distinguish normal and tumor plasma cells. The expression of cytoplasmic kappa and lambda light chains provides confirmation of clonality based on light chain restriction [27,36]. One such example of representative flow cytometry plots from peripheral blood of a diagnosed case of MM is depicted in Fig. 2. The usual sensitivity of flow cytometry assay is 10^−4^ to 10^−5^, however, with next-generation flow cytometry the sensitivity of multicolor flow assay may reach 10^−6^. There are certain limitations of flow cytometry-based assessment of CTPCs, like the lack of standardized protocols related to variations in the processing of samples, heterogeneous antibody panels, number of cells acquired for analysis which may also depend upon the volume and quality of the bone marrow aspirate withdrawn, difficulties in the intracellular staining for light chain analysis and expertise in data analysis. All these parameters contribute to the sensitivity of the assay and hence variations in the results across the laboratories [55].

## Next Generation Sequencing (NGS)

NGS for detection of clonotypic V(D)J immunoglobulin (Ig) rearrangement is the most recent and also one of the most sensitive techniques available, with detection of 1 tumor cell per 10^6^ BM cells. In addition to CPCs, circulating nucleic acid (cell-free DNA) is also a promising target for NGS and provides information on tumor load and response to therapy. It is feasible and better than the already existing assays, enabling accurate and specific index clone assessment and future tracking of all rearrangements in a patient sample [56].

The technique primarily utilizes the Ig heavy chain (IGH) gene rearrangements which occur during early B cell development. The combination of the germline variable (V), diversity (D), and joining (J) genes, results in the creation of unique V(D)J sequences. The functional V(D)J sequences encode for antigen-binding regions which by virtue of combinatorial diversity are specific for each lymphocyte. Later in development, somatic hypermutation adds to the diversity of the antigen-binding areas. The IGH locus contains 6 IGHJ, 27 IGHD and 38–46 functional IGHV genes [56]. The process of rearrangement involves trimming by exonuclease and random addition of nucleotides by terminal deoxynucleotidyl transferase (TdT) at the D-J and V-D-J junctions, forming the N-diversity regions before ligation. The rearranged V-D-J junction codes for the third complementarity-determining region (CDR3) and is the most diverse region of the V domain. The diversity of Ig molecules attained in the end is expected to be in the order of 10^12^ due to the large spectrum of potential rearrangements. Each rearrangement is independent and distinct, with a minimal chance that two unrelated cells would share the same sequence. As a result, a group of lymphocytes showing the same rearrangement will represent a clonal population [56].

However, depending on the depth (coverage) of sequencing, there may be interlaboratory variances in test sensitivity that are also linked with NGS-based techniques. Furthermore, these molecular techniques are not generally available, and their repeatability across laboratories is not properly established. Additionally, the sequencing/analysis time exceeds the clinically relevant timeframe, plus the significant cost despite the recent heavy cut off, makes this unpopular. The clinical impact of NGS-based assessment of CPCs in plasma cell neoplasms is currently not known and deserves thorough investigations [51,57].

Overall, the recent advancement in technologies, especially multi-color flow cytometry or next generation flow cytometry and the next generation sequencing has revolutionized the sensitivity to detect tumor plasma cells. The NGS presently remains the most sensitive technique, approaching detection sensitivity levels of 10^−6^ or below. However, the technique is available only in a few advanced referral laboratories and is inaccessible to most patients in developing and under-developed countries. Multi-color flow cytometry is a comparatively more easily available technique and with refined protocols and acquisition of more than 5 million events has shown detection sensitivities approaching those of NGS. Standardization across platforms and also amongst different laboratories is a tedious process due to numerous variables affecting the final results. Comparative studies between NGS and MFC have been carried out on minimal residual disease detection in MM patients on treatment [8,58]. Studies by Medina et al. [59,60] showed an excellent correlation between the two techniques for the detection of minimal residual disease in their cohort of patients MM. Similar findings of a very good correlation were noted by Oliva et al. [61] for the detection of minimal residual disease in patients MM in their phase II FORTE trial. The study revealed a remarkable prognostic concordance with hazard ratios in MFC-MRD and NGS-MRD-negative *vs*.-positive patients of 0.29 and 0.27 for progression-free survival and 0.35 and 0.31 for overall survival, respectively (*p* < 0.05). During maintenance, 4-year progression-free survival was 91% and 97% in 1-year sustained MFC-MRD-negative and NGS-MRD-negative patients (10^−5^), respectively, and 99% and 97% in 2-year sustained MFC-MRD-negative and NGS-MRD-negative patients, regardless of treatment received [61].

Table 1 provides a comparison between the techniques commonly used for the assessment of CTPCs. Detection of tumor cells at such high sensitivity at various time points during the course of therapy may eventually become part of patient care and likely to be used in therapeutic decisions. However, the validation and standardization of assays across platforms and the inter-laboratory comparability is an ongoing process.

**Table 1:** Comparison between techniques used for assessment of CTPCs

	Cytology	MFC	NGS
Sensitivity	10^−2^	10^–4^ to 10^−5^	10^−6^
Quantitative	Yes	Yes	Yes
Turnaround time	1–2 h	2–3 h	>7 days
Requirement of diagnostic sample	No	No	Yes
Need of fresh sample	Yes	Yes	No
Detection principle	Morphology	DfN	IgH-V(D)J rearrangement
Relative cost	Low	Intermediate	High

Note: DfN: difference from normal.

## Clinical Significance of CPCs

### CPCs and monoclonal gammopathy of undetermined significance (MGUS)

MGUS represents a condition where a monoclonal protein is detectable in serum and/or urine of an individual, however, there is no evidence of MM, amyloidosis, macroglobulinemia, or other related plasma cell or lymphoproliferative neoplasms [62]. MGUS is primarily an asymptomatic state with generally a low rate of progression to symptomatic neoplastic disease. The presence of circulating myeloma cells in individuals with MGUS has been studied, especially over the last two decades, and their burden in peripheral blood has been associated with the rate of progression of MGUS to overt MM.

Kumar et al. [53] studied the presence of plasma cells in the peripheral blood of 325 individuals with MGUS. CPCs were detected in 19.4% of their subjects. In individuals with the presence of CPCs, the median progression-free survival (PFS) was 138 months, whereas the median for PFS was “not yet reached” for those without CPCs. In addition, the individuals with CPCs had a median overall survival of 160 months which was shorter than the median survival of “not yet reached” at the last follow-up for individuals without CPCs. The study also evaluated various other factors like age, hemoglobin level, serum creatinine, serum albumin, serum ß2-microglobulin levels, M protein concentration, type of immunoglobulin, and presence of CPCs. The results of a univariate analysis note that higher M protein concentration, a non-IgG subtype of the involved immunoglobulin, and the presence of CPCs, were the only three factors to be significant predictors for progression. The combination of a load of CPCs with a higher M protein concentration and the M-protein of a non-IgG subtype identified a subset of individuals with MGUS who were at a higher risk of progressing to overt MM. The author suggested a scoring system based on these three variables to stratify the individuals with MGUS for their likelihood of progression to MM. The persons with higher risk of progression required closer monitoring. These individuals might become eligible for clinical studies testing new preventive medicines [53]. The data clearly show that the presence of CPCs was indicative of poor outcomes in terms of both PFS and OS.

### CPCs and SMM

SMM is defined as clonal PC infiltration in bone marrow comprising 10% or more of the total cellularity, and/or presence of serum M protein of 3 g/dL or more, in the absence of end-organ damage attributable to the proliferation of neoplastic plasma cells [57]. Due to a lack of data regarding the progression of the disease to the final malignant phase, patients mostly remain under observation without any early therapeutic support. This increases the mortality of around 20% of the patients who are at high risk of getting into the malignant phase within the first 2 years of disease. Also, there is the likelihood of serious end organ damage with malignancy, hence the observational approach may not always be beneficial [24]. The above factors partly contributed to considering the evaluation of CPCs as a prognostic indicator in patients of SMM by many groups.

Witzig et al. [36] studied a cohort of 57 individuals with SMM using slide-based immunofluorescence procedures that identified clonal plasma cells in the peripheral blood by microscopic examination of their morphology and the light chain expression. Within one year of screening for circulating clonal plasma cells, 16 (28%) patients progressed to MM and required treatment, whereas the other 41 patients remained stable. CPCs were noted at baseline in 10 out of the 16 (63%) patients who progressed within 12 months. In contrast, only four out of 41 (10%) of the stable patients had initial documentation of CPCs. Furthermore, the median time to progression to active MM in the patients with CPCs was 0.75 years. The progression rates for one & two years of time-period were 64.3% and 78.5%, respectively. In contrast, patients who did not show CPCs had a median time to progression of 2.5 years, with progression rates of 11.6% & 35.7% within one & two years, respectively. The authors concluded that the detection of CPCs was vital and may aid in the identification of patients having active MM when the rest of the parameters suggest SMM. The absence of CPCs in SMM may indicate a relatively stable disease without immediate treatment requirements [36]. Overall, the frequency of presence of CPCs is higher in patients who progress to active MM. In addition, the load of CPCs could identify the patients with the likelihood of progression in the absence of any indicators of higher risk like end organ damage.

Bianchi et al. [24] studied a cohort of 91 SMM patients. The authors defined a sub-group comprising 15% of patients who revealed a higher burden of CPCs, i.e., >5000 × 10^6^/L and/or >5% CPCs per 100 cytoplasmic Ig-positive peripheral blood mononuclear cells. This group of patients had a substantially rapid progression to active MM in comparison to the patients who had a lower load of CPCs, (12 months *vs*. 57 months of PFS), and was independent of other known risk factors for progression to MM. Most significantly, those with a high burden of CPCs had a 71% chance of progression during the first two years after diagnosis, in comparison to a 25% chance of progression in the rest of the patients with lower levels of CPCs. Similar findings were observed with overall survival, where the high-burden CPC group *vs*. the lower-burden CPC group showed OS of 49 *vs*. 148 months, respectively. The authors concluded that CPCs can be a suitable biomarker that can justify early therapeutic intervention in the absence of any end organ damage. A high burden of CPCs indicates impending active disease and also the presence of radiologically and clinically occult distant disease foci. The authors also observed that CPCs paired with M protein size may build a strong risk stratification model [24]. Overall, this study indicates that the higher load of CTPCs in PB is associated with poorer outcomes in terms of PFS and OS.

Foulk et al. [62] studied a cohort of 85 intermediate/high-risk SMM patients. CPCs were detected in 93.7% of cases at baseline. The load of CPCs at baseline was substantially higher in patients who ultimately progressed to active MM during the study period. The authors found that although the levels of M protein, free light chain ratio, and percentage of plasma cells in bone marrow also indicated disease progression, these parameters did not achieve a level of statistical significance. The study concluded that a well-standardized CPC-assay, in the context of predicting disease progression in patients of SMM, may have direct implications for the management of the disease, such as triggering more advanced diagnostic work-up or starting therapy sooner [62]. The study found that the load of CPCs is a stronger predictor of MM than conventional markers.

### CPCs and MM

MM is an advanced stage of malignancy amongst the spectrum of plasma cell neoplasms. It is characterized by the proliferation of neoplastic plasma cells in the bone marrow which frequently leads to the invasion of adjacent bone, resulting in the typical lytic bone lesions and pathological fractures. The common clinical features include anemia, hypercalcemia and renal failure [57].

CPCs are commonly noted in patients of MM at the time of diagnosis, especially with the use of high-sensitivity assays. There is substantial literature available on significance in patients of MM.

Gonsalves et al. [18] studied a cohort of 157 patients of MM. Multi-colour flow cytometry assay detected CPCs in 54% of their patients at diagnosis and the rest constituted the cohort with absence of CPCs. The follow-up revealed that neither group reached a median overall survival (OS), however, the survival was significantly less for the patients with the presence of “any level” of CPCs when compared with those showing the absence of detectable CPCs at baseline. Patients with CPCs had a 2- & 3-year OS of 76% and 67%, respectively, compared to 91% and 87% for patients without CPCs. The authors also observed that the number of CPCs detected by flow cytometry was an independent prognostic factor in patients with newly diagnosed MM treated with novel agents. Additionally, the study revealed that the presence of >400 CPCs of all WBCs, identified a subset of patients who had a shorter overall survival and also a shorter time-interval to the next therapy. The cut-off of >400 CPCs in their cohort seemed even better in predicting this high-risk group than the traditional prognostic markers like high risk cytogenetic and high ISS stage. The high burden of CPCs did correlate with high-risk cytogenetics as well as increased proliferation. Further, it indicated that this approach improved the ISS staging for the identification of a smaller subset of patients with a poorer outcome. It was concluded that the load of CPCs in newly diagnosed MM patients was a powerful predictor of early relapse from therapy and mortality. As a result, these findings may have consequences for modifications in the present criteria for risk-determination in MM, as well as may need an update in the practice of risk-adapted treatment protocols [18].

Foulk et al. [62] studied a cohort of 166 newly diagnosed (ND) MM patients. The authors used FISH on sorted PCs for analysis of CPCs, which were noted in 98% of their patients of MM. The overall disease burden in patients which included the percentage of plasma cells in the bone marrow, levels of serum M protein and ISS stage, correlated with the CPC counts. The authors found that the CPC counts significantly reduced from the baseline counts, in patients who responded to therapy and achieved remission. Also, patients with clonal plasma cell counts of ≥100 at the time of remission showed reduced survival. The authors also proposed that the stratification of patients according to CPC counts may assist in using remission CPC counts as a measure for minimal residual disease and also may be used as a surrogate endpoint for relapse in clinical trials [62].

Tembhare et al. [55] used MFC to analyze CTPCs in a cohort of 141 NDMM patients at the time-of diagnosis, and subsequently at two time points, that is after completion of three cycles of therapy [PBMRD1 (peripheral blood measurable residual disease 1)] and after completion of six cycles (PBMRD2). The study revealed CTPCs in 76.6% of their patients at baseline. The CTPCs were detectable in 44% of patients at the first post-therapy time-point (PBMRD1) and in 34.4% at the second time-point (PBMRD2). Patients with ≥0.01% CTPCs showed shorter median PFS and OS. However, a longer PFS was noted in patients with detectable CTPCs at baseline but undetectable CTPCs at further time-points of therapy. Patients who showed high-risk cytogenetics, RISS-II/III, R2ISS of intermediate & high-risk, were found to have greater levels of CTPCs. The authors also found that the undetectable combined PBMRD (PBMRD1 and PBMRD2) outperformed the serum-immunofixation-based response. The authors concluded that CTPCs evaluated at diagnosis and further PBMRD are good non-invasive biomarker for prognostication of NDMM [55].

Gupta et al. [54] carried out a sequential assessment of CPCs, at baseline and at 6 months of therapy, in a small cohort of 21 newly diagnosed MM patients, using a ten-color (eleven-antibody) flow cytometry assay. The study quantified CTPCs and circulating normal plasma cells (CNPCs), separately, at both time points. CTPCs were found in 76% and 33% of the patients at baseline and at 6 months, respectively. The load of CTPCs, which included percentage, absolute counts per microliter of peripheral blood, and the proportion of CTPCs out of all circulating plasma cells (CTPCs + CNPCs), were associated with the presence of lytic lesions, plasmacytomas, expression of CD56 and CD81, Chr1p32 deletion, and absence of very good partial response (VGPR). Conversely, the load of CTPCs was lower in patients with concomitant amyloidosis. Interestingly, CNPCs were significantly higher in female patients and were lower in patients with hypoalbuminemia and thrombocytopenia [54].

Gonsalves et al. [63] studied a cohort of 556 newly diagnosed MM. All patients in R-ISS stage I or R-ISS stage II who had ≥5 CTPCs/μL were re-categorized to R-ISS stage IIB. The authors noted that the median time to the next therapy and overall survival in patients re-categorized to R-ISS stage IIB was lesser (21 and 45 months) when compared to patients in R-ISS stage I (40 months and not reached) and R-ISS stage II (30 and 72 months) and was similar to patients with R-ISS stage III (20 and 47 months). The load of CTPCs retained its adverse prognostic significance in a multivariate analysis of the outcomes. Similar results were seen in the study by Xia et al. [64]. The authors sub-categorized their patients of R-ISS stage II into CTPC-low and CTPC-high and found that R-ISS I, R-ISS II with CTPC-low, R-ISS II with CTPC-high, and R-ISS III had median progression free survival of 41 months, 30 months, 19 months and 16 months. The introduction of CTPCs levels into R-ISS demonstrated more robust discrimination of prognosis of newly diagnosed cases of MM, especially in the patients presenting in R-ISS stage-II [63]. The findings of the above two studies clearly indicate that the load of CTPCs could be integrated into existing clinical workflow.

In the last decade, there have been a lot of studies on CPCs that tried to give a cutoff to indicate the favorable or poor outcome. Table 2 summarizes the key results of these studies.

**Table 2:** Summary of recent studies on CPCs in NDMM

	Gonsalves et al. [18]	Vagnoni et al. [31]	Gonsalves et al. [63]	Han et al. [65]	Galieni et al. [66]	Garcés et al. [67]	Bertamini et al. [68]	Kostopoulos et al. [69]	Tembhare et al. [55]
Study design	Retrospective	Prospective	Retrospective	Retrospective	Retrospective	Prospective	Prospective	Prospective	Prospective
Cohort type	NDMM	NDMM	NDMM	NDMM	NDMM	NDMM	NDMM	NDMM	NDMM
Cohort size	157	104	566	108	168	374	474	550	141
Median 65 Age (yrs.) (range)		72 (45–86)	66 (27–95)	63 (36–84)	71.5 (43–90)	57	57	68 (29–92) 55(27–82)	
Method for CPC detection	MFC	MFC	MFC	MFC	MFC	NGF	NGF	NGF	MFC
CTPC cut off value	>400 CPCs/150,000 events	41 CPCs/50,000 events	>400 CPCs/150,000 events	CPCs >0.105%	>1 CPCs (present/absent)	>0.01%	>0.07%	≥0.02%	≥0.01%
Key points	>400 CPCs/150,000 events indicate higher plasma cell proliferation, adverse cytogenetics, lesser OS.	Standard risk patients with >41CPCs have poorer outcomes.	>5 CPC/μL picks up a subgroup of patients in RISS II with poorer prognosis.	>0.105% clonal CPCs with RISS III fall into ultra-high risk groups.	OS and PFS in RISS II patients were lower in those having >1CPCs.	Patients with undetectable CTCs had exceptional PFS regardless of complete remission and MRD status.	>0.07% CTCs have shorter PFS and OS.	Patients without CTCs showed unprecedented outcomes (5-year PFS and OS: 83% and 97%, respectively). MRD-negativity was less frequent if CTCs were ≥0.02% at diagnosis.	CTPC ≥ 0.01% was independently associated with poor PFS & OS. PBMRD at any time point was independently associated with poor PFS.

Note: **CPCs**—Circulating plasma cells; **MFC**—Multiparametric flow cytometry; **NDMM**—Newly diagnosed MM; **NGF**—Next generation flow; **OS**—Overall survival; **PFS**—Progression free survival.

All the above studies in MM indicate that the higher load of CTPCs at diagnosis or any time point during the course of therapy indicate poor outcomes. In addition, higher CTPCs also correlated with many of the conventional prognostic markers. It is also noted that its inclusion in existing scoring systems may enhance their efficacy in identifying patients with poorer outcomes.

Importantly, the prognostic significance of CTPCs is likely to vary depending on the detection method and patient cohort. The load of CTPCs generally increases from the early stages of MGUS to symptomatic MM. Hence, cohorts of different sub-categories of plasma cell neoplasms are likely to provide distinct cut-offs of levels of CTPCs for assessing outcome parameters. A sample bias in studies involving fewer subjects of MGUS and/or smoldering myeloma is more likely because of the asymptomatic nature of these conditions and hence robust cut-offs for these entities require larger screening cohorts and longer follow up durations. For symptomatic myeloma, variations amongst different studies for the cut-off value of the levels of CTPCs at the time of diagnosis are likely to produce variations in the prognostic significance of CTPCs. These variations in values are likely due to different technologies used to detect CTPCs. Morphological assessment of CPCs is easiest but with very low sensitivity as compared to MFC and NGS. A cut off of ≥5% CPCs on morphological assessment is presently used to categorize patients into the poorer outcome entity of plasma cell leukemia as compared to MM. Even for sophisticated techniques like MFC, the sensitivity varies a lot and depends on the methodology of sample processing, the number of cells acquired for analysis, the number and type of antibodies used in the panel, experience and expertise of the flow cytometrist involved in analysis. The above issues are reflected in the results of levels of CTPCs in different studies. In addition, for assessment of CTPCs during the course of therapy, important variables like time points at which the samples are analyzed, type of chemotherapeutic regimen administered, whether patients have undergone hematopoietic stem cell transplant or not, and the duration of follow up of patients, are very likely to influence the prognostic information provided by CTPCs.

### CPCs and autologous stem cell transplantation (ASCT) in MM

Studies have shown that the presence of CPCs prior to ASCT is indicative of both reduced PFS and OS and is independent of the depth of response evaluated at the end of induction [21,70]. In newly diagnosed patients of MM undergoing upfront ASCT, a reduction in load of CPCs by proteasome inhibitor (PI) and/or immunomodulator (IMiD)-based induction therapy, leads to an improved PFS and OS [23] (Table 3).

**Table 3:** Summary of recent studies on CPCs in MM-patients undergoing ASCT

	Dingli et al. [71]	Chakraborty et al. [21]	Chakraborty et al. [23]
Study design	Prospective	Retrospective	Prospective
Cohort type	MM prior to ASCT	MM prior to ASCT	MM with CPCs at diagnosis and prior to ASCT
Cohort size	246	840	247
Median age (yrs)(range)	57 (30–74)	61 (24–76)	62 (25–76)
Method of CPCs detection	MFC	MFC	MFC
Key points	Patients with CMCs prior to transplant have lesser CR & shorter OS & TTP.	Patients with CPCs at transplant showed a negative prognostic impact for depth of response, PFS, OS irrespective of high-risk cytogenetics, stage at diagnosis & pre-transplant response.	Serial assessment of CPCs before induction and before ASCT was independent of ISS stage, high-risk cytogenetics, LDH at diagnosis, and pre-transplant response.

Note: **CMCs**—Circulating myeloma cells; **MFC**—Multiparametric flow cytometry; **OS**—Overall survival; **TTP**—Time to progression; **PFS**—Progression free survival.

Dingli et al. [71] studied a cohort of 246 MM patients undergoing ASCT. They found circulating clonal plasma cells in 95 of their patients. Although the complete response (CR) rates after transplantation were similar for their patients with or without CPCs, however, the overall survival and the time to progression were significantly shorter in patients with the presence of CPCs prior to transplant. The authors also noted that the CPCs remained independent of cytogenetic and disease status at the time of transplantation. The authors also proposed a scoring system using both CPC counts and cytogenetics in combination. They concluded that CPCs at the time of ASCT were an independent prognostic factor and in combination with cytogenetic is likely to provide a powerful scoring system that could stratify the patients into risk-groups in a better way and also guide management [71].

Chakraborty et al. [21] studied a cohort of 840 MM patients and utilized a six-color flow cytometry-assay to detect CPCs prior to ASCT. The authors noted CPCs in 162 (19.3%) patients in their cohort. Patients harboring CPCs at the time of ASCT had a greater incidence of having high-risk cytogenetic abnormalities in comparison to those without CPCs. The stringent complete response (sCR) rates after ASCT were significantly lower in patients with detectable CPCs prior to transplant as compared to those without CPCs (15% *vs*. 38%, respectively). Similar results of significantly poor PFS (15.1 *vs*. 29.6 months) and OS (41 *vs*. not reached) were noted in their patients with and without detectable CPCs, respectively. The authors concluded that sequential quantification of CPCs during the course of disease should be incorporated in clinical trials to study the clonal evolution and kinetics of CPCs, and its impact on disease outcomes [21].

Chakraborty et al. [23] studied a cohort of 247 MM patients undergoing ASCT. The authors carried out a sequential evaluation of CPCs at the time of diagnosis and pre-transplant. They categorized patients into three groups i.e., CPCs (−/−) who had no circulating CPCs at both time points (n = 117), CPCs (+/−) had CPCs at diagnosis followed by complete eradication post induction therapy (n = 82), and CPCs (+/+) had CPCs both at the time of transplant and continued presence of cells or emergence of new cells post-induction therapy (n = 48). Post-transplant stringent CR rates were significantly higher in the first group and were found to be 32%, in CPCs (−/−), 30% in CPCs (+/−) and 12% in CPC (+/+) groups. The PFS and OS after transplant were significantly different amongst the three groups with CPCs (−/−) group showing the best outcome. The authors concluded that monitoring for CPCs before induction therapy and prior to transplant was predictive of survival in newly diagnosed MM and should be incorporated into clinical trials [23].

## Conclusions

CTPCs are a subset of tumor cells that exit into peripheral blood as a result of the dynamic interactions between the tumor plasma cells and BM microenvironment, the most important being the CXCR4 & CXCL12 interaction. The role of CTPCs in the context of the entire spectrum of plasma cell neoplasms is of great interest to scientists and physicians in this field. There is already an intense effort amongst different laboratories for inter-platform and inter-laboratory standardization of CTPCs-detection and accurate quantification, both using multicolor flow cytometry or next-generation sequencing. Presently, there is sufficient clinical data available to conclude that assessing the load of the CTPCs is an independent prognostic factor across the spectrum of plasma cell neoplasms, especially monoclonal gammopathy of unknown significance, smoldering myeloma, and symptomatic MM. Sequential monitoring of CTPCs in individuals with MGUS or SMM seems to have potential in capturing those who are progressing faster to symptomatic MM and are potential candidates for further work-up and possibly also for early initiation of therapy. There is evidence to indicate that assessment of the load of CTPCs could be integrated into existing clinical workflow and its incorporation is likely to enhance the prognostic value of R-ISS risk stratification and thus help in better management of patients. In addition, the sequential monitoring of CTPCs in patients on therapy may provide even greater inputs on response to therapy and/or course of disease and may be considered as a surrogate marker representing the overall burden of myeloma cells in the body at that time-point of course of therapy. Blood monitoring is less invasive than bone marrow procedures and unlike bone marrow aspirate samples, it is not affected by patchy distribution or hemodilution. Peripheral blood monitoring is also more suited for more frequent monitoring as compared to bone marrow MRD analysis. Future analyses of larger cohorts of patients, including those who are transplant-ineligible, and other cohorts like patients treated with anti-CD38 monoclonal antibodies or CAR-T cell therapy, along with a longer follow-up duration, may help to identify a definitive cut-off for CTPCs for better prognostication. This will be the next step for the implementation of CTPCs in comprehensive staging systems and risk-adapted therapeutic approaches. It is expected that BM aspirates will remain the gold-standard specimen for the diagnosis and genetic characterization of MM patients. However, given the prognostic significance of CTPCs, it may be suggested that quantification and genetic characterization of CTPCs may emerge as a complementary approach for non-invasive risk-stratification of MM patients, with the possibility of avoiding invasive BM examination at certain time points during the clinical course of selected patients.

## Data Availability

The data that support the findings of this study are available from the corresponding author upon reasonable request.
